# Seasonal variation in particulate organic carbon sequestration in subarctic and subtropical gyres of the western North Pacific

**DOI:** 10.1038/s41598-026-43514-8

**Published:** 2026-03-23

**Authors:** Yoshihisa Mino, Chiho Sukigara, Kazuhiko Matsumoto, Tetsuichi Fujiki, Minoru Kitamura, Masahide Wakita, Chisato Yoshikawa, Makio C. Honda

**Affiliations:** 1 Institute for Space-Earth Environmental Research, Nagoya University, Nagoya, Japan; 2 Japan Agency for Marine-Earth Science and Technology, Yokosuka, Japan; 3 Mutsu Institute for Oceanography, Japan Agency for Marine-Earth Science and Technology, Mutsu, Japan

**Keywords:** Nitrogen isotope ratios, Particulate organic carbon flux, Net primary productivity, Carbon sequestration, Biogenic opal and CaCO_3_ fraction, Biological carbon pump, Carbon cycle, Marine chemistry

## Abstract

The ocean’s biological carbon pump regulates climate by transferring a portion of surface-fixed CO_2_ to the deep ocean through sinking particulate organic carbon (POC). Although this flux is strongly attenuated in the twilight zone, the seasonal controls on attenuation remain poorly understood. We examined seasonal variations in POC flux, the nitrogen isotope ratio of sinking particulate nitrogen (δ^15^N_sink_), and mineral composition using sediment traps observations at subarctic (K2) and subtropical (S1) stations in the western North Pacific. POC sequestration efficiency at 500 m [Seq_(500)_ = POC flux/NPP] was quantified, with net primary productivity (NPP) reconstructed from δ^15^N_sink_ using an empirical framework. Seq_(500)_ remained nearly constant at K2 (7.4–8.1%) but varied substantially at S1 (3.4–6.5%). At K2, CaCO_3_ and opal contents exhibited complementary seasonal patterns, whereas S1 showed pronounced variability primarily in CaCO_3_. We propose that mineral composition modulates aggregate settling velocity and adhesive strength, thereby regulating POC attenuation through fragmentation processes. These findings indicate that seasonal shifts in surface ecosystem structure influence the physical properties of sinking aggregates and ultimately control the fate of NPP in the twilight zone.

**Supplementary Information:**

The online version contains supplementary material available at 10.1038/s41598-026-43514-8.

## Introduction

The ocean serves as a major sink for atmospheric carbon dioxide (CO_2_), playing a central role in the global carbon cycle. A key mechanism underlying this sink is the biological carbon pump (BCP), which exports carbon fixed by surface photosynthesis to the deep ocean via sinking organic particles, thereby sequestering CO_2_ on decadal to centennial timescales^1^. As with primary production, the magnitude of BCP-driven carbon sequestration varies substantially among oceanic regions^2^. Improving its representation in models is therefore critical to reducing uncertainties in future carbon cycle projections.

The annual global export flux of particulate organic carbon (POC) from the euphotic zone is estimated at ~ 5 to 10 Gt C, yet less than 10% ultimately reaches depths below 1000 m^3,4^. Rapid attenuation of sinking POC flux in the twilight zone, commonly described by a power-law depth function^5^, exhibits pronounced spatial variability. Proposed mechanisms include microbial remineralization, zooplankton grazing, particle disaggregation or fragmentation^6–8^, modulated by temperature, oxygen, productivity, and phytoplankton community structure^9–13^. The “ballast effect,” whereby biogenic minerals enhance POC export and transfer efficiencies^14^, has received particular attention. Ballast minerals increase aggregate density and settling velocity^15^ and may reduce microbial degradation through physical protection^16^, thereby influencing remineralization length scales^17^. However, the functional roles of CaCO_3_, opal, and lithogenic minerals remain debated^18^, and relationships between mineral composition and settling velocity vary among regions and seasons^19–21^. Recent estimates suggesting that microbial remineralization accounts for a smaller fraction of POC flux attenuation than previously assumed^22^ further emphasize the potential importance of physical fragmentation processes. Together, these findings highlight the need for an integrative framework linking particle composition, ecosystem structure, and attenuation dynamics.

**Fig. 1:**
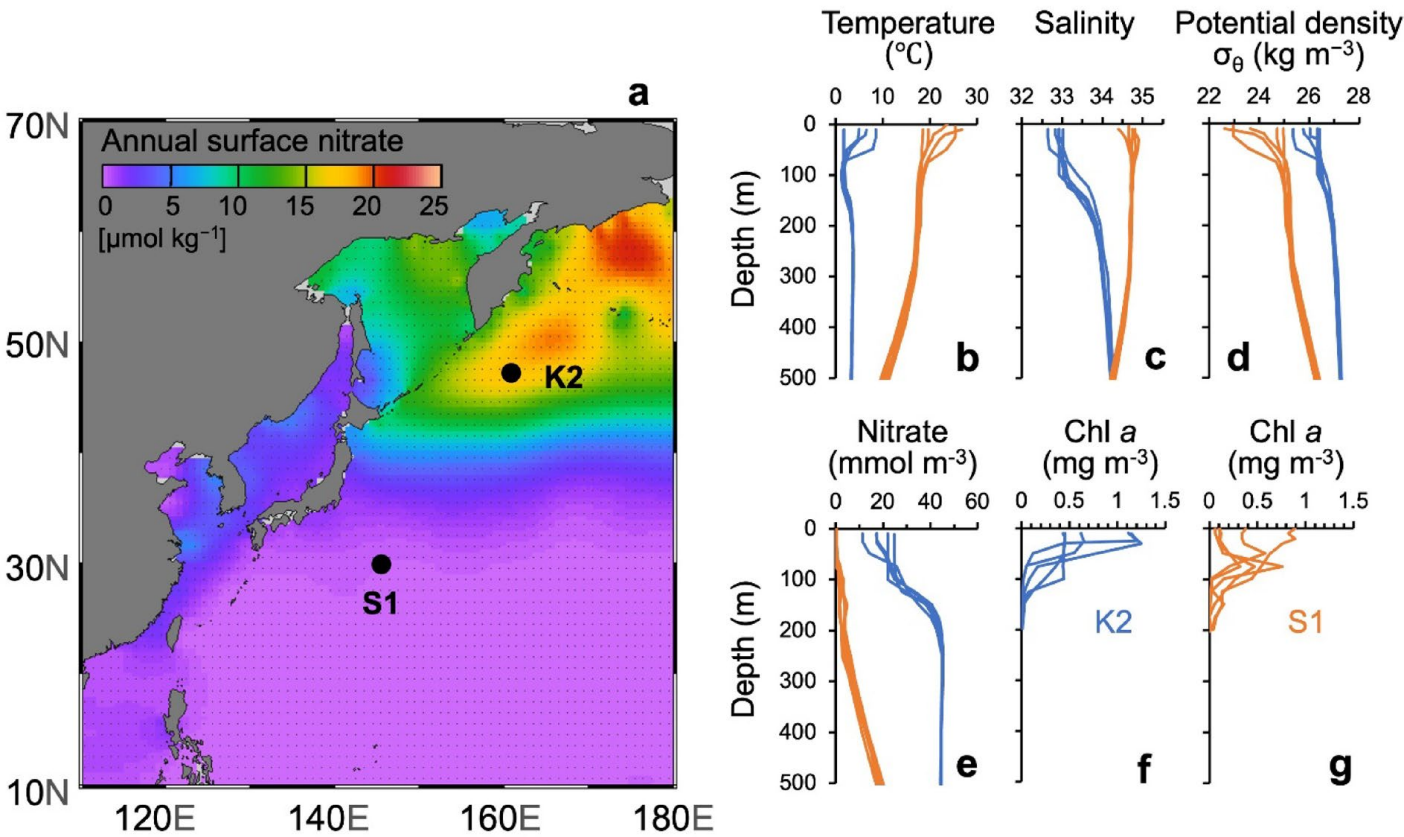
(**a**) Locations of time-series stations in the subarctic (K2) and subtropical (S1) regions, together with annual surface nitrate concentrations from World Ocean Atlas 2023 Data^27^, created using Ocean Data View 5.7.2 (https://odv.awi.de), and vertical profiles of parameters in the upper water column at K2 (blue) and S1 (orange) observed during the five cruises in 2010–2012: (**b**) water temperature; (**c**) salinity; (**d**) density anomaly σ_θ_ (potential density − 1000 kg m^−3^); (**e**) nitrate; (**f, g**) chlorophyl a concentrations.

Here we investigate two contrasting regions of the western North Pacific: the subarctic gyre (station K2) and the subtropical gyre (S1; Fig. 1). Using sinking particle samples collected by moored sediment traps (MSTs) at 500 m between 2010 and 2014, we reconstruct a time series of the fraction of net primary production (NPP) transported to the mesopelagic zone, defined as the POC sequestration efficiency at 500 m (Seq_(500)_ = POC flux/NPP)^23^, and compare its seasonal variability between the two sites. It has been reported that K2 and S1 differ markedly in phytoplankton community structure and bloom phenology—a diatom-dominated summer bloom at K2 and a multi-taxa winter bloom at S1^24,25^—as well as in the mineral composition of exported particles (opal-dominated at K2 versus CaCO_3_-rich at S1)^26^. These contrasting characteristics provide a natural framework for examining controls on mesopelagic POC transfer efficiency.

To resolve seasonal variability in Seq_(500)_, an NPP time series with comparable temporal resolution is required. We therefore use the nitrogen isotopic composition (δ^15^N) of sinking particles as a proxy for NPP. At K2, δ^15^N of particulate nitrogen (PN) collected at 100–200 m by drifting sediment traps (DSTs) was negatively correlated with incubation-based NPP estimates^28^, providing the basis for establishing an empirical relationship between MST particle δ^15^N and contemporaneous NPP. For S1, a direct δ^15^N–NPP relationship has not been explicitly evaluated; however, δ^15^N of sinking particles at 200 m decreases during winter blooms^29^, suggesting that 500 m trapped particle δ^15^N likely reflect productivity variability. We assess whether MST particle δ^15^N can serve as a proxy for NPP and POC export at both sites and integrate evidence on sinking velocity, vertical δ^15^N profiles, and mineral composition to propose a site-specific mechanism linking ballast minerals, particle dynamics, and seasonal variability in POC sequestration efficiency.

## Materials and methods

### Site characteristics

Station K2 (47°N, 160°E; bottom depth 5240 m) is located in the western subarctic gyre of the North Pacific (Fig. 1), within a high-nutrient, low-chlorophyll region where surface nitrate persists throughout the year. Phytoplankton growth is seasonally limited by iron or light availability^24,30,31^, resulting in a diatom-dominated summer bloom with surface chlorophyll a (Chl. a) concentrations exceeding 1 mg m^−3^^28^. Surface nitrate concentrations typically range from 10 to 25 mmol m^−3^, peaking in early spring following winter convective mixing (mixed layer depth ~ 150 m) and reaching minima in late summer^32^. Although nitrate declines during stratified summer conditions due to biological uptake, depletion is incomplete because of iron limitation. In contrast, surface ammonium concentrations increase in summer (> 1 mmol m^−3^), reflecting active remineralization, and decrease in winter (~ 0.2 mmol m^−3^) due to enhanced nitrification below the euphotic zone^28,33^.

Station S1 (30°N, 145°E; bottom depth 5970 m) is located in the western North Pacific subtropical gyre and serves as a low-nutrient counterpart to K2^34^. From May to November, strong stratification limits nutrient supply, with surface nitrate falling below 0.1 mmol m^−3^ and often becoming undetectable. Surface Chl. a remains low (< 0.1 mg m^−3^), and the phytoplankton community is dominated by with *Prochlorococcus*^25^. A subsurface Chl. a maximum (~ 0.5 mg m^−3^) typically forms between 50 and 90 m depth, just above the nitracline and is composed of diverse taxa, including prymnesiophytes (coccolithophores), chlorophytes, and chrysophytes. Episodic increases in subsurface chlorophyll maximum occur in association with mesoscale eddy activity that uplifts nitrate-rich subtropical mode water^29,35^. During winter (December–March), monsoon-driven mixing deepens the mixed layer (> 200 m), elevates surface nitrate (~ 0.4 mmol m^−3^), and supports phytoplankton blooms (surface Chl. a up to ~ 1.0 mg m^−3^) composed of mixed taxa, including diatoms, chlorophytes, prymnesiophytes, and chrysophytes^25,36^. Ammonium concentrations remain low year-round (0.02–0.07 mmol m^−3^), indicating tight coupling between regeneration and biological uptake within the euphotic zone^37^.

### Data used

We analyzed MST data (particle fluxes, δ^15^N, and chemical composition) obtained from 500 m at K2 and S1 from February 2010 to May/June 2014 as part of the K2S1 project^34^. The initial K2 MST dataset (February 2010–June 2012) has been reported previously^28^; here we extend the record by an additional two years to better resolve seasonal variability while reducing the influence of interannual fluctuations.

For S1, we used unpublished 500 m MST data. Although 200 m trap data from the same mooring system has been published^29^, these data were not used here because the winter mixed layer at S1 often exceeds 200 m. If particulate organic matter collected by the 200 m trap were remineralized, solubilized, or transformed into inorganic forms within layers shallower than the deepest mixed layer, these materials could potentially be returned to the surface by deep mixing. Therefore, not all POC trapped at 200 m can be considered definitively sequestered from the surface ocean, making it unsuitable for the purpose of this study. Moreover, because this study focuses on regional differences in surface-origin particle behavior affecting POC flux attenuation, it is appropriate to compare POC sequestration efficiency at the same depth (500 m) at both K2 and S1.

Seasonal NPP data measured using a ^13^C tracer method at both sites from 2010 to 2013 were used to examine relationships with MST particle δ^15^N. These NPP data have been published previously^36^, and methodological details are provided therein.

### Sediment trap deployment and chemical analyses of samples

Time-series, bottom-tethered sediment traps (Nichiyu-Giken SMD26S-6000, Japan; McLane Mark VII-21, USA) were deployed at 500 m and recovered annually between 2010 and 2014. Sampling intervals ranged from 12 to 18 days.

Sample preservation (10% buffered formalin), swimmer removal, splitting, filtration, drying, and pulverization followed established protocols^38^. Total mass flux was determined gravimetrically. Concentrations of Al, Si, and Ca were measured using inductively coupled plasma atomic emission spectrometer (Optima 3300DV, Perkin‐Elmer, USA). POC and PN in the samples were measured using an elemental analyzer (Flash2000, Thermo Fisher Scientific, USA) after 24-h of hydrochloric acid fumigation to remove inorganic carbon. Concentrations of organic matter, biogenic opal, CaCO_3_, and lithogenic material were estimated following Honda et al.^38^. δ^15^N of trapped particles (δ^15^N_sink_) was determined using an elemental analyzer (EA1110) coupled to a continuous flow isotope-ratio mass spectrometer (Delta Plus, Thermo Fisher Scientific, USA) and calibrated against the IAEA-N-1 international standard. Analytical precision was better than ± 0.2‰.

### Data processing

**Fig. 2:**
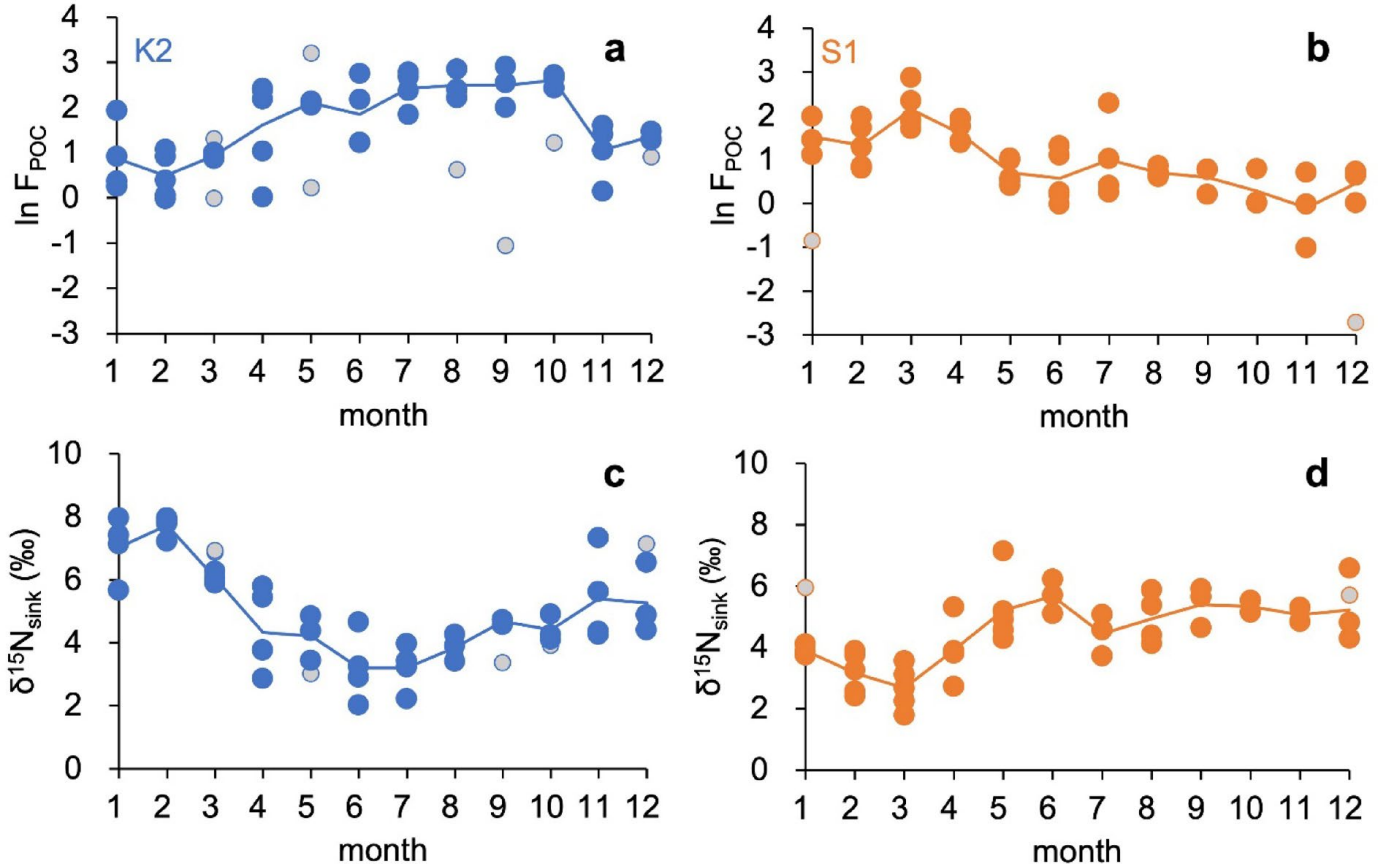
Variations in natural log-transformed particulate organic carbon flux (F_POC_) at 500 m and the nitrogen isotopic composition of sinking particles (δ^15^N_sink_) at stations K2 (blue, left panels: **a**, **c**) and S1 (orange, right panels: **b**, **d**), obtained from sediment trap deployments during 2010–2014. Solid lines connect the monthly means for the entire deployment period. Gray circles in panels (**a**) and (**b**) indicate F_POC_ outliers for each month, defined by the 1.5-fold interquartile range rule; the corresponding δ^15^N_sink_ values for these outliers are indicated by the same symbols in panels (**c**) and (**d**) Note: the seasonal variation of absolute F_POC_ data is provided as Supplementary Fig. S2.

Monthly POC flux and δ^15^N_sink_ values were used for analyses. Normality was evaluated using the Shapiro–Wilk test (α = 0.05). Raw POC flux values were non-normally distributed at both sites (*p* < 0.05), whereas log-transformed values met the normality assumption (*p* > 0.05). δ^15^N_sink_ values were normally distributed at both sites (*p* > 0.05, Supplementary Fig. S1). Outliers in the natural log–transformed POC flux dataset were identified using the 1.5-fold interquartile range criterion and excluded (Fig. 2a–b).

To quantify seasonal variability, we calculated the relative anomaly of monthly POC flux (F) from the deployment-period mean flux (F_ave_). The ln-transformed relative anomaly, hereafter referred to as rel- F_POC_, was defined as:

1 $${\mathrm{rel}} - {\mathrm{F}}_{{{\mathrm{POC}}}} = {\text{ln }}\left[ {\left( {{\mathrm{F}} - {\mathrm{F}}_{{{\mathrm{ave}}}} } \right)/{\mathrm{F}}_{{{\mathrm{ave}}}} + {1}} \right]$$

The constant “+1” ensures positive arguments for logarithmic transformation. Expressing variability in relative rather than absolute terms has the advantage of allowing correction for potential under-collection biases associated with shallow MST deployment (see next section) when reconstructing δ^15^N-based POC flux.

An empirical regression between δ^15^N_sink_ and rel-F_POC_ was established and used to reconstruct monthly POC flux anomalies from δ^15^N_sink_. Absolute monthly fluxes were then obtained by combining reconstructed anomalies with independently estimated annual mean POC fluxes at 500 m. Representative annual mean 500 m fluxes were derived from depth-dependent power-law attenuation relationships reported by Honda^26^, using attenuation exponents *b* of 0.64 for K2 and 0.90 for S1 (see caption of Table 2). These relationships are based on vertically resolved flux profiles measured with surface-tethered DSTs and deep MSTs. These flux data in the upper layer are supported by independent dissolved carbon and nutrient budget analyses^32^. The resulting annual mean 500 m POC fluxes were 23.6 mg C m^−2^ d^−1^ (K2) and 17.2 mg C m^−2^ d^−1^ (S1).

## Results

### Annual mean particle properties, NPP, and POC sequestration efficiency

This study is based on particle samples collected using bottom-tethered conical MSTs deployed at 500 m. Such traps are known to potentially under-collect particles due to hydrodynamic biases^39^. During the deployment period, the measured mean POC fluxes were 6.7 mg C m^−2^ d^−1^ at K2 and 3.4 mg C m^−2^ d^−1^ at S1 (Table 1), representing 28% and 20%, respectively, of independently estimated annual mean fluxes at 500 m (23.6 and 17.2 mg C m^−2^ d^−1^; Table 2; see “Data processing” section). In subsequent analyses, we assume that under-collection was temporally uniform and therefore focus on relative seasonal variability rather than absolute flux magnitude.

**Table 1:** Mean fluxes, chemical compositions, C:N molar ratios, and nitrogen isotopic composition (δ^15^N_sink_) of trapped particles at 500 m at Stations K2 and S1 during the entire sediment trap deployments from 2010 to 2014. Note: POC, particulate organic carbon; OM, organic matter; Opal, biogenic opal; LM, lithogenic matter.

	Flux (mg m^−2^ d^−1^)		Fraction (%)				(mol mol^−1^)	(‰)
	Total mass	POC	OM	Opal	CaCO_3_	LM	C:N	δ^15^N_sink_
K2	114.1 ± 112.0	6.7 ± 5.7	19.0 ± 7.7	43.7 ± 19.3	34.6 ± 18.4	2.8 ± 2.3	8.1 ± 1.6	5.0 ± 1.7
S1	32.3 ± 40.0	3.4 ± 3.2	33.1 ± 16.3	4.5 ± 2.5	59.0 ± 15.4	3.4 ± 1.7	9.1 ± 3.3	4.5 ± 1.2

**Table 2:** Annual mean net primary productivity (NPP), POC flux (F_POC_), and sequestration efficiency (Seq_(500)_), based on the results of preceding studies and modeled in the present study using δ^15^N_sink_ values at 500 m. ^a^NPP data are from Matsumoto et al.^36^. ^b^Martin curve-F_POC_ was estimated using power-law functions fitted to upper-layer POC flux data (60–200 m) and deep flux data (4810 m), as proposed by Honda^26^. The site-specific regression equations with depth z (m) are follows, Y = − 0.64X + 4.55 at site K2 and Y = − 0.90X + 4.76 at site S1, where Y and X represent the natural logarithms of F_POC_(z) and z/60, respectively. ^c^Both NPP and F_POC_ were calculated by substituting monthly mean δ^15^N_sink_ data at 500 m into empirical relationships between δ^15^N_sink_ and measurements of NPP and relative POC flux, as shown in Fig. 3d–f.

	Measurements-based			δ^15^N_sink_-based		
	NPP^a^ (mg C m^−2^ d^−1^)	Martin curve-F_POC_^b^ (mg C m^−2^ d^−1^)	Seq_(500)_ (dimensionless)	NPP^c^ (mg C m^−2^ d^−1^)	F_POC_^c^ (mg C m^−2^ d^−1^)	Seq_(500)_ (dimensionless)
K2	292	23.6	0.081	280	22.2	0.079
S1	303	17.2	0.057	307	14.5	0.047

**Fig. 3:**
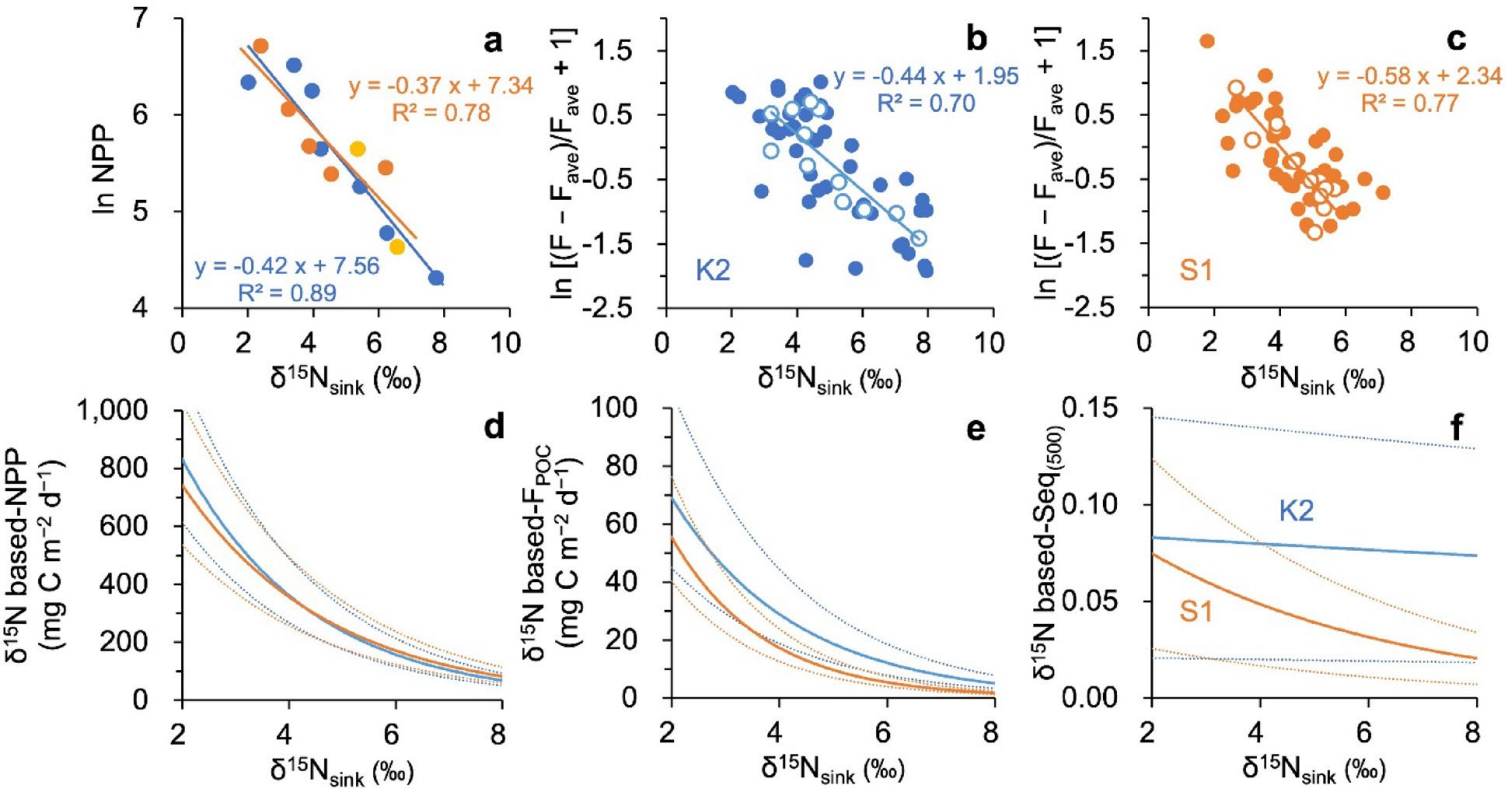
Relationships between δ^15^N_sink_ (‰) and (**a**) natural log-transformed net primary productivity (ln NPP; dimensionless), and the relative deviation from the mean F_POC_ (dimensionless) at K2 (**b**) and S1 (**c**). The relative deviation was calculated as [(F − F_ave_)/F_ave_ + 1], where F and F_ave_ represent the individual POC flux and the mean flux over the entire sediment trap deployment period, respectively (presented as Eq. 1 in text). In panel **a**, blue and orange circles represent K2 and S1 data, respectively; yellow circles denote S1 data with δ^15^N_sink_ lagged by one month relative to the NPP measurement. Open circles in panels (**b**) and (**c**) indicate monthly mean values. Solid blue and orange lines in panels (**a**)–(**c**) show the regression lines for K2 and S1, respectively, all with *p* values < 0.01. In panels (**b**) and (**c**), regressions are based on monthly means from 2010 to 2014. The lower panels (**d**–**f**) illustrate the modeled relationships between δ^15^N_sink_ and (**d**) NPP (mg C m^−2^ d^−1^), (**e**) F_POC_ (mg C m^−2^ d^−1^), and (**f**) POC sequestration efficiency to 500 m depth (Seq_(500)_; dimensionless). F_POC_ in panel **e** was calculated assuming annual mean fluxes, Martin curve-F_POC_, of 23.6 mg C m^−2^ d^−1^ at K2 and 17.2 mg C m^−2^ d^−1^ at S1 (see “Data processing” section). Dashed lines in panels (**d**)–(**f**) indicate the upper and lower bounds of the modeled values, reflecting estimation uncertainties. The NPP data in panel **a** are from Matsumoto et al.^36^.

In contrast, particle composition is less sensitive to collection efficiency^38^. At K2, mean mass fractions were 44 ± 19% biogenic opal, 35 ± 18% CaCO_3_, 19 ± 8% organic matter, and 2.8 ± 2.3% lithogenic material. At S1, particles were dominated by CaCO_3_ (59 ± 15%) and organic matter (33 ± 16%), with lower contributions from opal (4.5 ± 2.5%) and lithogenic material (3.4 ± 1.7%; Table 1). These contrasting compositions reflect the canonical “silica ocean” (diatom-dominated) and “carbonate ocean” (coccolithophore-dominated) regimes^40^. Organic C:N molar ratios averaged 8.1 ± 1.6 at K2 and 9.1 ± 3.3 at S1, and mean δ^15^N_sink_ values were 5.0 ± 1.7‰ and 4.5 ± 1.2‰, respectively (Table 1); inter-site differences were not statistically significant (Welch’s t-test, t(69) = – 1.86, *p* = 0.07 for C:N; t(93) = 1.84, *p* = 0.07 for δ^15^N).

Annual mean NPP was comparable between sites (292 vs. 303 mg C m^−2^ d^−1^, Table 2)^36^, although depth-integrated Chl. a was higher at K2 (42.1 vs. 34.4 mg m^−2^). Combining NPP with independently estimated 500 m POC fluxes yielded mean POC sequestration efficiencies, Seq_(500)_, of 0.081 at K2 and 0.057 at S1 (Table 2), indicating ~ 1.4-fold higher efficiency at K2.

### Seasonal variability of POC flux and δ^15^N_sink_

Monthly POC flux at 500 m varied between 14–262% (K2) and 11–520% (S1) of the deployment-period mean, corresponding to rel-F_POC_ values of −1.9 to +1.0 and −2.2 to +1.6, respectively. At K2, elevated rel-F_POC_ occurred during the stratified period (July–October), whereas at S1 maxima occurred during the well-mixed period (January–April). These patterns were broadly consistent with available direct NPP observations, which showed maxima of 677 mg C m^−2^ d^−1^ (July 2011) at K2 and 826 mg C m^−2^ d^−1^ (February 2011) at S1.

δ^15^N_sink_ ranged from 2.0–8.0‰ at K2 to from 1.8–7.1‰ at S1 (Fig. 2c–d). Lower δ^15^N_sink_ values coincided with periods of high NPP and elevated rel-F_POC_—during summer stratification at K2 and winter mixing (mixed layer depth > 100 m) at S1—consistent with previous site-specific observations Mino et al.^28^ and Mino et al.^29^. At S1, newly analyzed 500 m δ^15^N_sink_ closely matched contemporaneous 200 m values, with mean differences comparable to analytical precision (Supplementary Fig. S3) and no detectable seasonal lag. This indicates that most particles collected at 500 m were exported from shallower depths and reached 500 m within approximately one month.

### Proxy performance of δ^15^N_sink_ and estimation of monthly Seq_(500)_

**Fig. 4:**
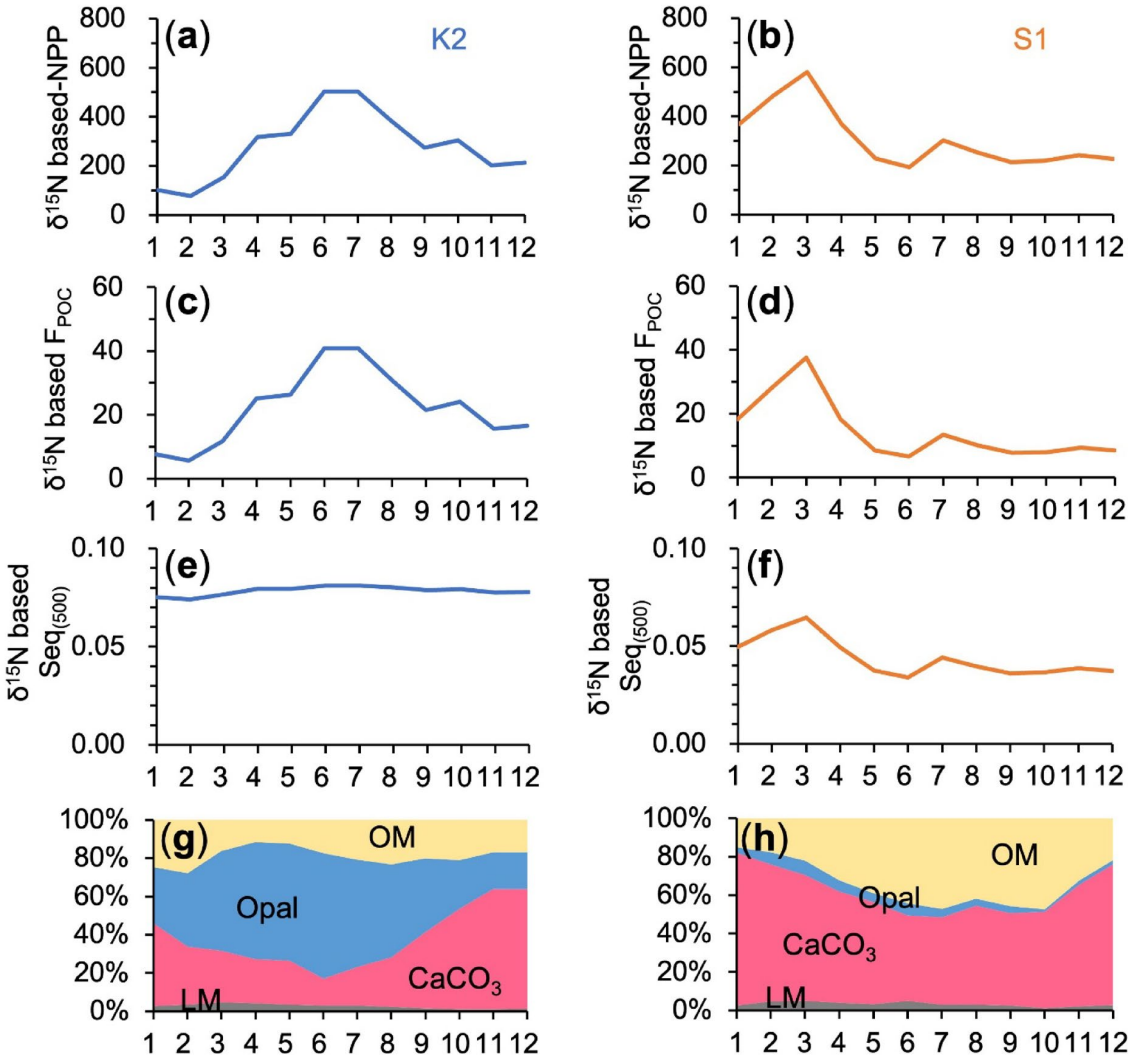
Seasonal variations in δ^15^N_sink_-based monthly means of NPP (mg C m^−2^ d^−1^) (**a**–**b**), F_POC_ (mg C m^−2^ d^−1^) (**c**–**d**) and Seq_(500)_ (**e**–**f**) at 500 m, and chemical composition (Organic Matter, Opal, CaCO_3_, and Lithogenic Materials) (**g**–**h**) of trapped particles at K2 (left panels) and S1 (right).

Significant negative correlations were observed between δ^15^N_sink_ and ln-transformed NPP at both sites (R^2^ = 0.89, *p* = 0.001 at K2; R^2^ = 0.78, *p* = 0.008 at S1; n = 7), with no significant difference in regression slopes (*p* = 0.37; Fig. 3a). Similarly, δ^15^N_sink_ was negatively correlated with rel-F_POC_ (R^2^ = 0.54, *p* < 0.001 at K2 and 0.47, *p* < 0.001 at S1 for individual months), and correlations strengthened when monthly climatological means were used (R^2^ = 0.70, *p* < 0.001 at K2 and 0.77, *p* < 0.001 at S1; Fig. 3b–c). Using these regressions, monthly NPP, POC flux, and Seq_(500)_ were reconstructed from δ^15^N_sink_. Estimated NPP ranged from 77–503 mg C m^−2^ d^−1^ at K2 to 193–580 mg C m^−2^ d^−1^ at S1. Reconstructed POC flux ranged from 5.7–40.8 mg C m^−2^ d^−1^ at K2 to 6.5–37.5 mg C m^−2^ d^−1^ at S1. Monthly Seq_(500)_ varied narrowly at K2 (0.074–0.081) but more broadly at S1 (0.034–0.065; Fig. 4a–f). Because Seq_(500)_ varied monotonically with δ^15^N_sink_ (Fig. 3d–f), maxima and minima occurred in June and February at K2 and in March and June at S1, respectively.

Annual means derived from δ^15^N-based reconstructions were 280 and 307 mg C m^−2^ d^−1^ for NPP, 22.2 and 14.5 mg C m^−2^ d^−1^ for POC flux, and 0.079 and 0.047 for Seq_(500)_ at K2 and S1, respectively (Table 2). Thus, POC sequestration efficiency remained substantially higher at K2, by a factor of ~ 1.7.

## Discussion

### Seasonal drivers of δ^15^N_sink_

Particle δ^15^N at K2 was lower in summer and higher in winter, whereas the opposite pattern was observed at S1, with an overall seasonal amplitude of ~ 6‰ at both sites. If δ^15^N_sink_ reflects variations in the isotopic composition of nitrogen sources, these contrasting seasonal cycles ultimately arise from regional differences in the δ^15^N dynamics of regenerated ammonium. At K2, progressive nitrate consumption from winter to summer increases nitrate δ^15^N through isotopic fractionation during uptake^28^. Surface nitrate δ^15^N increased by ~ 5.5‰ from February to July during the MST deployment period^41^. In parallel, ammonium δ^15^N increased markedly from summer (3.9–8.6‰) to autumn (13.4–13.7‰), indicating substantial ^15^N enrichment of regenerated NH_4_^+^. Assimilation of this enriched ammonium during winter mixing likely contributes to elevated winter δ^15^N_sink_.

In contrast, ammonium concentrations at S1 remain extremely low year-round because regeneration is closely balanced by biological uptake. Surface (0–100 m) NH_4_^+^ concentrations (0.02–0.07 mmol m^−3^) are an order of magnitude lower than suspended PN, the source of NH_4_^+^ regeneration^29^. Under near-complete consumption, isotopic fractionation during ammonium utilization becomes minimal, and regenerated NH_4_⁺ should approximate the δ^15^N of its source PN. Because most annual nitrogen input at S1 is supplied as subsurface nitrate^29^, PN and regenerated ammonium should inherit the seasonal δ^15^N evolution of nitrate, which increases as nitrate is progressively consumed. Although direct δ^15^N measurements of low-concentration nitrate are unavailable, this framework explains the summer enrichment and winter depletion observed in δ^15^N_sink_. Nitrogen fixation (δ^15^N ≈ −1‰^42^) may represent an additional low-δ^15^N source in oligotrophic waters. However, assuming subsurface nitrate δ^15^N of +4.5‰^29,43^ and steady-state conditions, the annual PN flux-weighted mean δ^15^N_sink_ (+3.9‰) implies that N_2_ fixation contributes only ~ 11% of total nitrogen supply at S1. While episodic inputs cannot be excluded, N_2_ fixation is unlikely to dominate the seasonal δ^15^N signal with a winter minimum.

Overall, seasonal differences in δ^15^N_sink_ reflect distinct nitrogen cycling regimes: asynchronous nitrate–ammonium isotopic dynamics at K2 versus broadly synchronous evolution of regenerated and new nitrogen at S1.

### Mechanisms underlying the negative δ^15^N_sink_–NPP relationship at both sites

At K2, the negative correlation between δ^15^N_sink_ and NPP (Fig. 3a) arises from the coupled effects of light availability and nitrification, as proposed by Mino et al.^28^. Briefly, during winter deep mixing, low light conditions suppress primary production, while nitrification below the euphotic zone produces ^15^N-enriched ammonium that can be entrained upward and assimilated (Supplementary Fig. S4). Enhanced reliance on this ^15^N-enriched ammonium by phytoplankton^44^ results in high δ^15^N_sink_ but low NPP. In contrast, during summer stratification improves light conditions and stimulates productivity, while photoinhibition of nitrifiers^45^ reduces the supply of ^15^N-enriched ammonium, leading to lower δ^15^N_sink_. Seasonal light forcing therefore regulates both productivity and nitrogen source δ^15^N, generating the observed inverse relationship.

At S1, phytoplankton growth is generally nitrogen-limited. Winter mixing injects nitrate into the surface layer, triggering blooms and elevated NPP^36,46^. Under high nitrate availability, phytoplankton preferentially assimilate ^14^NO_3_^−^^47^, producing isotopically light particles. As nitrate becomes progressively consumed, the residual pool becomes ^15^N-enriched, and lower-productivity periods yield higher δ^15^N particles. This seasonal isotopic trajectory, previously documented at 200 m^29^, is now also evident in 500 m (Fig. 2d) and produces the negative correlation with ln(NPP) (Fig. 3a). Although winter mixed layers at S1 often exceed 150 m, potentially imposing light limitation^48^, the lowest δ^15^N_sink_ values coincide with high NPP, implying that peak production occurs not under fully mixed conditions but during transient stratification events^49^. Mesoscale eddy activity south of the Kuroshio Extension frequently induces short-term surface stratification, creating shallow warm layers over nitrate-rich waters that support rapid phytoplankton growth^50,51^. Satellite observations of episodic blooms in this region are consistent with this mechanism^49^ and explain the close coupling between low δ^15^N_sink_ and elevated NPP.

Potential heterotrophic δ^15^N modification during sinking (e.g., trophic transfer and microbial remineralization via peptide-bond hydrolysis)^52–55^ appears limited. Amino acid–based trophic position (TP) analyses for K2 trap material showed little seasonal variation (TP ≈ 2.3 ± 0.1)^41^, and δ^15^N profiles of 100–200 m DST particles were vertically constant across seasons at both sites^56^. The close agreement between 200 and 500 m δ^15^N_sink_ at S1 (Supplementary Fig. S3) further supports negligible preferential ^14^N loss during transit. Seasonal variability in δ^15^N_sink_ thus primarily reflects surface nitrogen cycling rather than water-column processing, accounting for its strong correlation with NPP.

This empirically derived δ^15^N_sink_–NPP relationship provides an independent constraint on productivity at both sites. Satellite-based NPP estimates rely on surface chlorophyll retrievals^57^, which are limited at K2 by persistent summer fog^58^ (Supplementary Fig. S5), at S1 by subsurface chlorophyll maxima that are not directly detectable from space^59,60^. In this context, the δ^15^N_sink_-based approach offers a complementary, observation-based reconstruction of NPP with temporal resolution comparable to POC flux measurements. Notably, despite distinct nitrogen cycling regimes, the δ^15^N_sink_–ln(NPP) regressions are remarkably similar between sites. This convergence likely reflects comparable annual productivity and similar mean δ^15^N_sink_ values at 500 m (Table 1), although such relationships should not be assumed universal across oceanic regions.

### Seasonal variability of δ^15^N-based Seq_(500)_ and its linkage to ballast minerals

At both sites, δ^15^N_sink_ was negatively correlated with rel-F_POC_ (Fig. 3b–c), and combining this relationship with the δ^15^N_sink_–ln(NPP) regression yielded monthly estimates of Seq_(500)_. Although mean Seq_(500)_ was consistently higher at K2, seasonal variability was substantially larger at S1 (Fig. 4e–f). The ratio of maximum to minimum monthly Seq_(500)_ was 1.9 at S1 but only 1.1 at K2. Thus, at S1, POC produced during winter–spring blooms was transported to 500 m nearly twice as efficiently as during low-productivity months, whereas transfer efficiency at K2 remained nearly invariant. These contrasting patterns correspond to differences in ballast mineral composition. The combined contribution of CaCO_3_, opal, and lithogenic material ranged from 72–88% at K2 to 53–82% at S1 (Figs. 4g–h). The higher mean mineral fraction at K2 (81% vs. 67%; Table 1) is consistent with, and may partly explain, its ~ 1.7-fold greater annual Seq_(500)_. Moreover, seasonal variability in total mineral contribution was larger at S1 (~ 30%) than at K2 (~ 16%), paralleling the greater seasonal amplitude in Seq_(500)_.

However, enhanced mineral “protection” alone cannot explain these differences. The vertical invariance of particle δ^15^N between 100 and 200 m^56^ indicates that most PN flux attenuation—up to 65% at K2 and 59% at S1—occurred without significant ^15^N fractionation, implying dominance of physical fragmentation over selective remineralization. Agreement between 200 and 500 m MST δ^15^N_sink_ at S1 further supports minimal isotopic alteration during transit. Thus, regional differences in mineral-associated protection against microbial decay are unlikely to fully account for higher transfer efficiency at K2. Differences in settling velocity (SV) also do not provide a straightforward explanation. Seasonal SV measurements of 100 and 200 m DST particles showed faster annual mean sinking at S1 (63 ± 26 m d^−1^) than at K2 (31 ± 16 m d^−1^) despite lower total mineral fractions, a pattern attributed to higher CaCO_3_ content at S1 and lower temperatures (higher water viscosity) at K2^61^. If similar dynamics apply at 500 m, then the slower sinking at K2 would require proportionally lower POC decay rates to sustain its higher transfer efficiency.

We therefore propose that particle disaggregation is the primary driver of POC attenuation in the twilight zone at both sites. This interpretation is consistent with recent observations^22^ indicating that microbial respiration accounts for only ~ 20% of upper mesopelagic POC flux attenuation across multiple oceanic regions, with the remainder attributable largely to fragmentation processes. At K2, where fecal pellets substantially contribute to flux^62^, coprorhexy and coprophagy may further enhance disaggregation^63^. Resistance to fragmentation depends strongly on extracellular polymeric substances (EPS) and transparent exopolymer particles (TEP)^64–66^, which are linked to phytoplankton and bacterial biomass^67–69^. K2 exhibits higher mean chlorophyll concentrations than S1, suggesting greater EPS/TEP production and stronger aggregate adhesive strength. Although EPS-rich aggregates may reduce settling velocity due to lower density, enhanced adhesive strength would suppress fragmentation, thereby reducing POC loss during transfer and sustaining higher Seq_(500)_ at K2.

**Fig. 5:**
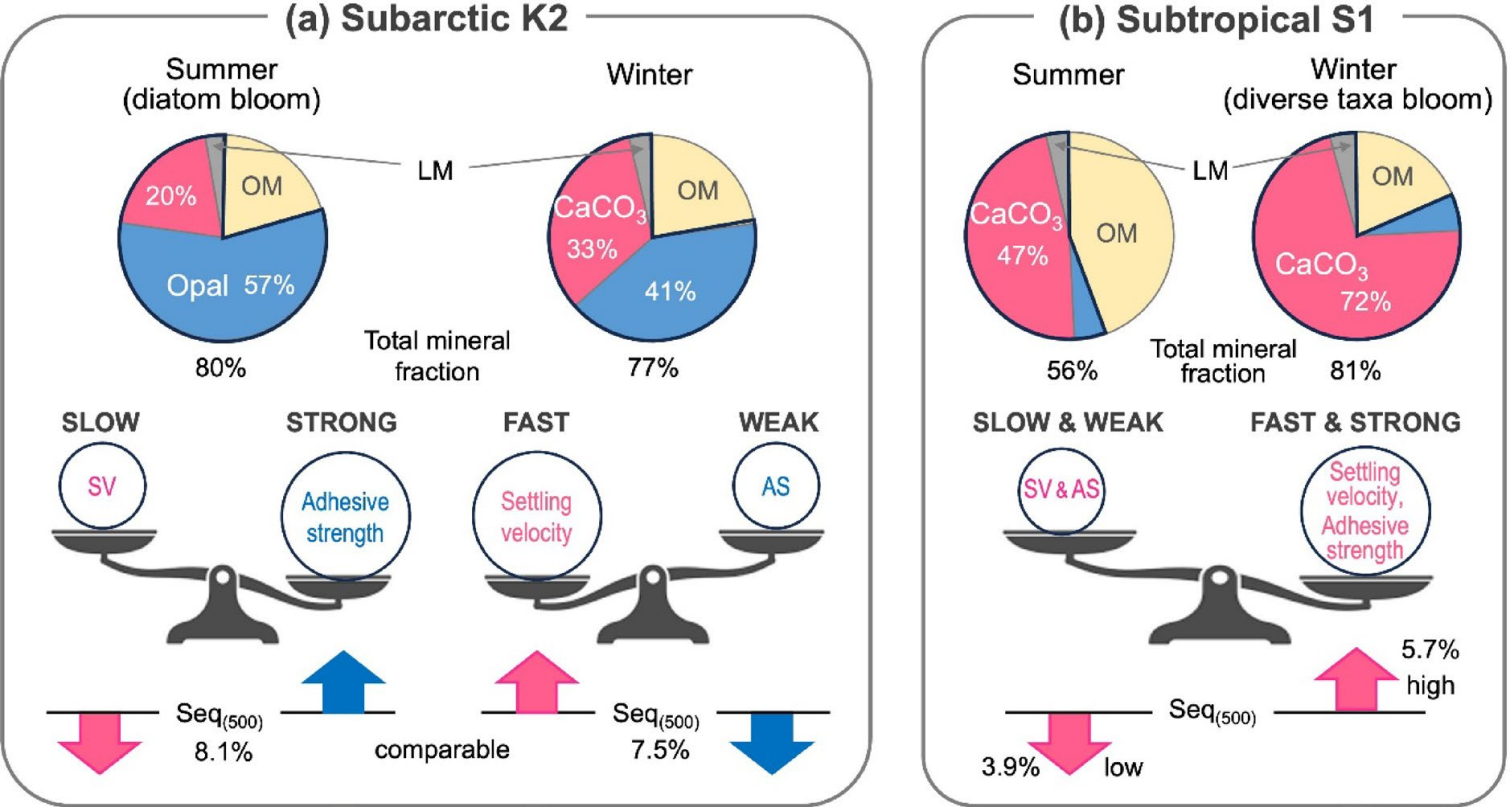
Proposed conceptual model of the seasonal relationships among chemical composition, physical properties—settling velocity (SV) and adhesive strength (AS)—of trapped aggregates, and POC sequestration efficiency at 500 m (Seq_(500)_) in subarctic K2 and subtropical S1. Fast SV and strong AS both positively influence Seq_(500)_, with their relative importance illustrated by the size and weight of the spheres placed on the upper pans of the balances shown at the center of each panel. The positive and negative seasonal effects of SV and AS on Seq_(500)_ are represented by arrows at the bottom (upward for positive, downward for negative). SV and AS are assumed to vary depending on the content of ballast minerals, particularly calcium carbonate (red) and opal (blue), with their respective influences indicated by the colors of sphere labels and arrows. (**a**) K2. During summer diatom blooms, the abundant extracellular polymeric substances (EPS) and transparent exopolymer particles (TEP) enhances the adhesive strength of settling aggregates. However, settling velocity decreases due to lower CaCO_3_ fraction. In winter, reduced diatom dominance and lower productivity limit the availability of EPS and TEP, weakening AS (downward blue arrow), while SV increases due to higher CaCO_3_ fraction (upward red arrow). These opposing seasonal trends in AS and SV counterbalance each other’s effects on POC sequestration efficiency, resulting in relatively stable Seq_(500)_ throughout the year. (**b**) S1. In winter, blooms composed of diverse phytoplankton, including coccolithophores, increase CaCO_3_ content and enhance SV. Simultaneously, greater availability of EPS and TEP strengthens AS. Conversely, both SV and AS decline during summer when productivity was lower, leading to higher Seq_(500)_ in winter compared to summer.

Seasonal mineral dynamics provide additional insight into the inter-site contrast. At K2, Opal% varied substantially (19–66%; Fig. 4g), broadly tracking seasonal phytoplankton biomass and diatom abundance (Supplementary Fig. S6). Under opal-rich summer bloom conditions, increased aggregate adhesive strength would be expected to enhance transfer efficiencies. Nevertheless, Seq_(500)_ remained nearly constant. Notably, CaCO_3_% and Opal% at K2 were nearly complementary, and their combined fraction was relatively stable (~ 78 ± 5%). If aggregate SV is primarily controlled by CaCO_3_%, then seasonal strengthening (or weakening) of adhesive strength associated with Opal% may have been offset by concurrent decreases (or increases) in SV. These opposing tendencies likely compensated for one another, stabilizing the seasonal balance between particle disaggregation and vertical transport, resulting in minimal net seasonal change in Seq_(500)_ (conceptualized in Fig. 5a). In contrast, at S1, CaCO_3_% increased to ~ 80% during winter blooms. In addition to strengthening aggregate adhesive strength, elevated CaCO_3_% likely accelerated settling velocity, jointly producing the pronounced winter peak in Seq_(500)_ (Fig. 5b).

### Export and transfer efficiencies linked to ballast minerals

**Fig. 6:**
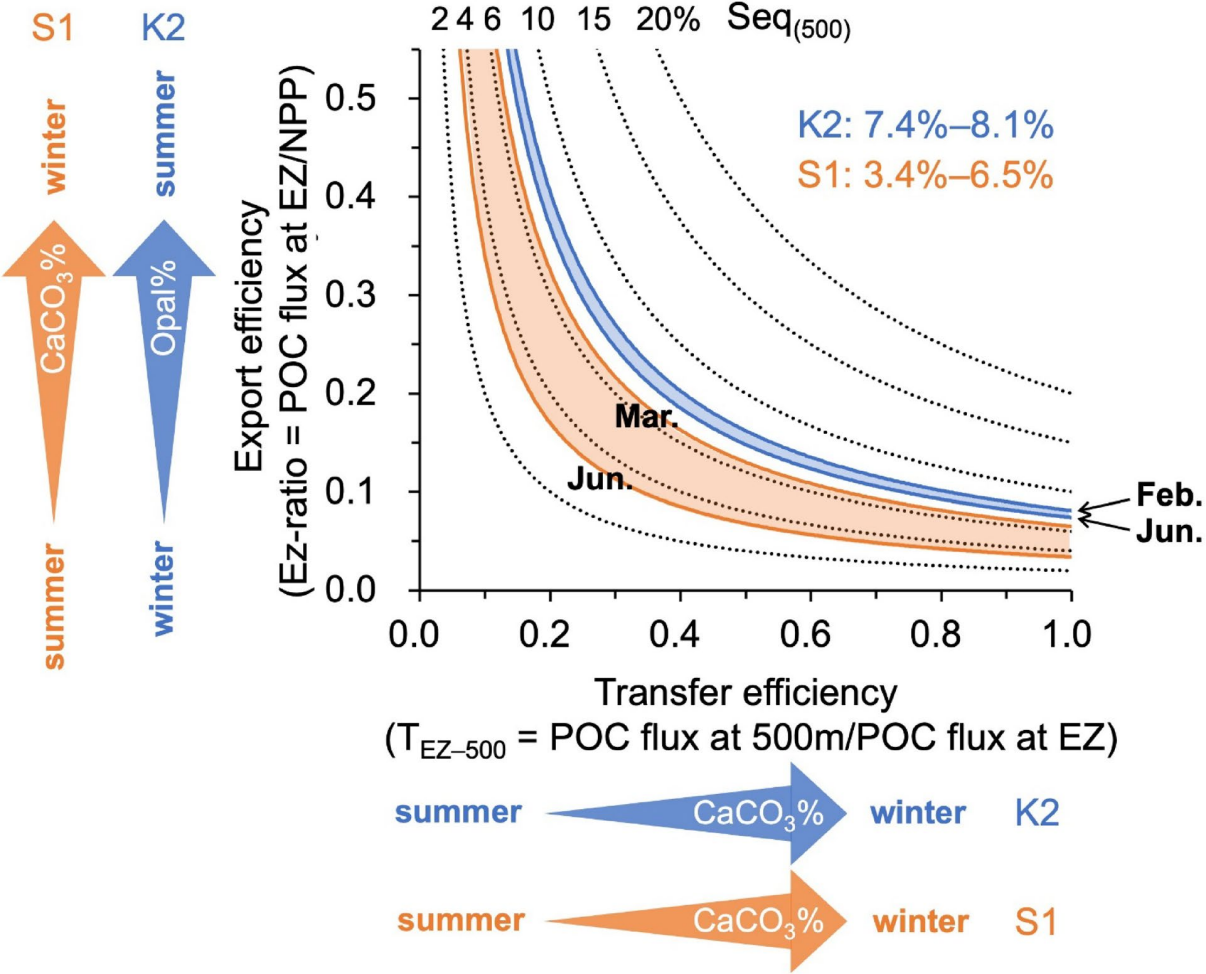
Seasonal relationships among export efficiency (Ez-ratio), transfer efficiency (T_EZ–500_), and ballast mineral composition at K2 and S1. The Ez-ratio represents the ratio of net primary production (NPP) to POC flux at the base of the euphotic zone (EZ), while T_EZ–500_ denotes the transmission efficiency of POC flux from below the EZ to 500 m. The range of Ez-ratio values (Y-axis) follows Buesseler and Boyd^70^. Contour lines (2–20%) indicate constant POC sequestration efficiency at 500 m, Seq_(500)_. Blue and orange shaded regions show the combinations of Ez-ratio and T_EZ–500_ consistent with the estimated seasonal ranges of Seq_(500)_ at K2 and S1, respectively. Arrows along the Y- and X-axes illustrate the hypothesized mineral controls, whereby seasonal increases in Opal% are associated primarily with enhanced export efficiency, whereas increases in CaCO_3_% are more strongly linked to enhanced transfer efficiency at K2. This framework highlights the distinct functional roles of ballast minerals in regulating carbon sequestration.

Seq_(500)_ can be expressed as the product of the POC export efficiency from the euphotic zone and the transfer efficiency to 500 m. The near-seasonal constancy of Seq_(500)_ at K2 suggests coordinated variation between these two efficiencies. Although disentangling their individual controls is challenging, patterns imply differential mineral influences: as illustrated in Fig. 6, export efficiency aligns more closely with Opal%, whereas transfer efficiency tracks CaCO_3_%. At S1, CaCO_3_% may influence both processes through its effects on settling velocity and aggregate adhesive strength. Previous syntheses by Buesseler and Boyd^70^ and Siegel et al.^71^ have emphasized the importance of jointly evaluating export and transfer efficiencies to understand regional and seasonal carbon sequestration. For example, high export (> 0.4) and near-unity transfer efficiencies during North Atlantic diatom blooms have been linked to opal-rich systems. However, the K2 results suggest a more nuanced relationship, in which opal and CaCO_3_ play distinct functional roles rather than exerting uniform ballast effects. Seasonal variability in mineral composition is tightly coupled to ecosystem structure and bloom timing, indicating that relationships between export/transfer efficiencies and aggregate composition are likely site-specific. Although our δ^15^N-based reconstructions do not provide absolute efficiency values, they reveal contrasting seasonal sequestration regimes at two western North Pacific sites with different nutrient limitations. This distinction is particularly relevant in the Pacific, where carbon sequestered into intermediate and deep waters remains isolated longer than in other basins^72^.

Our proposed framework relies on several assumptions. First, we assume that microbial remineralization contributes only modestly to flux attenuation, based on the isotopic constancy of sinking particles. While compound-specific isotope analyses^73^ would provide stronger constraints, independent studies also highlight the importance of fragmentation processes. Using source amino acids and trophic position estimates, Wojtal et al.^74^ demonstrated in the oligotrophic subtropical North Pacific that POC flux attenuation above 500 m is dominated by fecal-pellet disaggregation, with only minor contributions from solubilization and trophic remineralization. If such mechanisms are broadly applicable, they provide a mechanistic foundation for our interpretation. The concept of “non-selective preservation,” as evidenced by the compositional stability of sinking organic matter with depth^16,75^, further supports this view. Nonetheless, direct constraints on microbial processing within sinking aggregates—such as in situ measurements of remineralization rates^22^—remain necessary to fully evaluate this framework.

Second, if fragmentation rather than microbial remineralization plays a dominant role in flux attenuation, then particle physical properties become central in regulating transfer efficiency. In this context, we treat CaCO_3_% as a primary determinant of aggregate sinking velocity (SV), based on the observed positive correlation between CaCO_3_% and SV in DST particles at K2 and S1^61^. However, SV also depends on aggregate size and porosity^76^, and further in situ optical measurements^77,78^ are required to constrain these effects. While many studies suggest that denser CaCO_3_ provides more effective ballast than opal^79–81^, Honda^26^ proposed that opal may play a more important role in deep POC transfer at K2. Although we do not assume a direct physical control of opal on SV, we consider a positive relationship between Opal% and aggregate adhesive strength mediated by TEP production. Diatoms dominant at K2 are known to release abundant polysaccharides during active growth^82^, promoting TEP formation and enhancing adhesive strength. Nevertheless, quantitative constraints on natural aggregate adhesive strength remain limited^83–86^, and further investigation of aggregate formation and fragmentation dynamics is needed.

## Conclusions

We analyzed the seasonal variations in the δ^15^N_sink_ and POC flux at 500 m depth from sediment trap deployments at two contrasting sites in the western North Pacific—the subarctic K2 and the subtropical S1—to elucidate controls on carbon sequestration efficiency via the biological carbon pump.

At both sites, δ^15^N_sink_ exhibited a strong negative correlation with NPP and POC flux, with lower δ^15^N values during periods of high productivity (i.e., bloom seasons: summer at K2 and winter at S1). These seasonal patterns reflect site-specific limitations on phytoplankton growth and associated changes in the δ^15^N of nitrate and ammonium utilized in each region. Monthly POC sequestration efficiency at 500 m, Seq_(500)_, estimated from δ^15^N, remained nearly constant at K2 (7.4–8.1%) but varied almost twofold at S1 (3.4–6.5%).

To account for this contrast, we proposed a mechanism in which CaCO_3_ and opal fractions serve as proxies for key physical properties of sinking aggregates—namely sinking velocity and adhesive strength (i.e., resistance to fragmentation). This framework consistently explained the observed seasonal behavior of Seq_(500)_.

Our findings highlight that the physical properties of exported aggregates, shaped by phytoplankton biomass and community composition, exert strong control over deep-ocean carbon sequestration through their influence on particle dynamics during transit. In particular, we emphasize the role of aggregate fragmentation, a critical yet poorly constrained process in particle-based carbon cycle models^87^. Although further validation of the proposed linkage between aggregate adhesive strength and fragmentation is required, our results indicate that incorporating fragmentation dynamics into future BCP simulations will be essential for predicting changes in ocean carbon sequestration under evolving environmental conditions.

## Supplementary Information

Below is the link to the electronic supplementary material.

Supplementary Material 1

## Data Availability

The datasets used in the current study are available from the corresponding author on reasonable request.
