# Supplementary material for: Seasonal variation in particulate organic carbon sequestration in subarctic and subtropical gyres of the western North Pacific
Source: Sci Rep. 2026 Mar 23;16:14557. doi: 10.1038/s41598-026-43514-8 (PMC13153212; doi:10.1038/s41598-026-43514-8)
Supplement: Supplementary file 1 — Supplementary Material 1 [file 41598_2026_43514_MOESM1_ESM.pdf]

# Seasonal Variation in Particulate Organic Carbon Sequestration in Subarctic and Subtropical Gyres of the Western North Pacific

**Yoshihisa Mino[1*], Chiho Sukigara[2], Kazuhiko Matsumoto[2], Tetsuichi Fujiki[2], Minoru Kitamura[2], Masahide Wakita[3], Chisato Yoshikawa[2], Makio C. Honda[2]**

[1]Institute for Space-Earth Environmental Research, Nagoya University, Nagoya, Japan
[2]Japan Agency for Marine-Earth Science and Technology, Yokosuka, Japan
[3]Mutsu Institute for Oceanography, Japan Agency for Marine-Earth Science and Technology, Mutsu, Japan

Corresponding author: Yoshihisa Mino (mino.yoshihisa.i0@f.mail.nagoya-u.ac.jp)

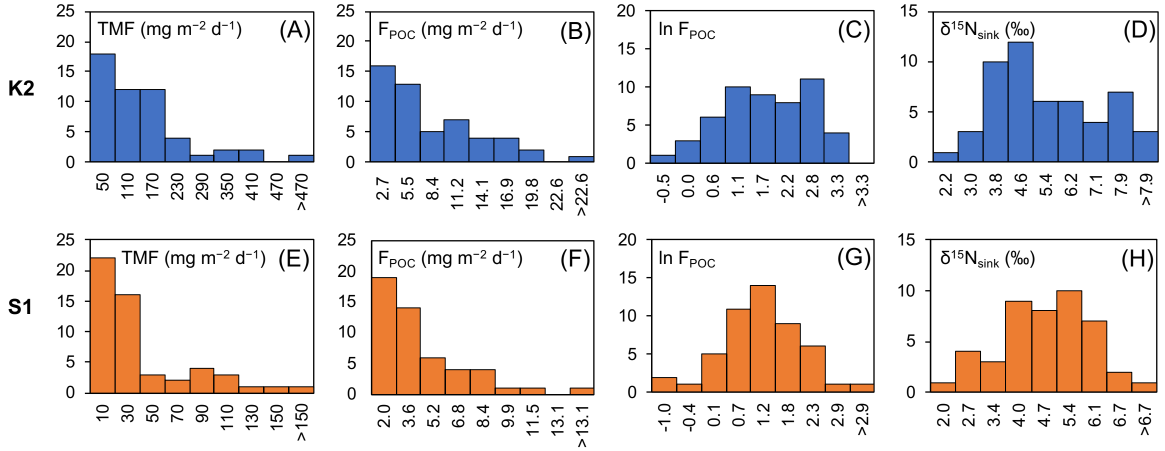


**Supplementary Figure S1.** Frequency distributions of (A, E) total mass flux, (B, F) particulate organic carbon (POC) flux, (C, G) natural log-transformed POC flux, and (D, H) nitrogen isotope ratios ($\delta^{15}N_{sink}$) of trapped particles at K2 (upper panels) and S1 (lower panels). Normality was evaluated using the Shapiro–Wilk test ($\alpha = 0.05$). Raw POC flux values were non-normally distributed at both sites ($p < 0.05$), whereas log-transformed values met the normality assumption ($p > 0.05$). $\delta^{15}N_{sink}$ values were normally distributed at both sites ($p > 0.05$).

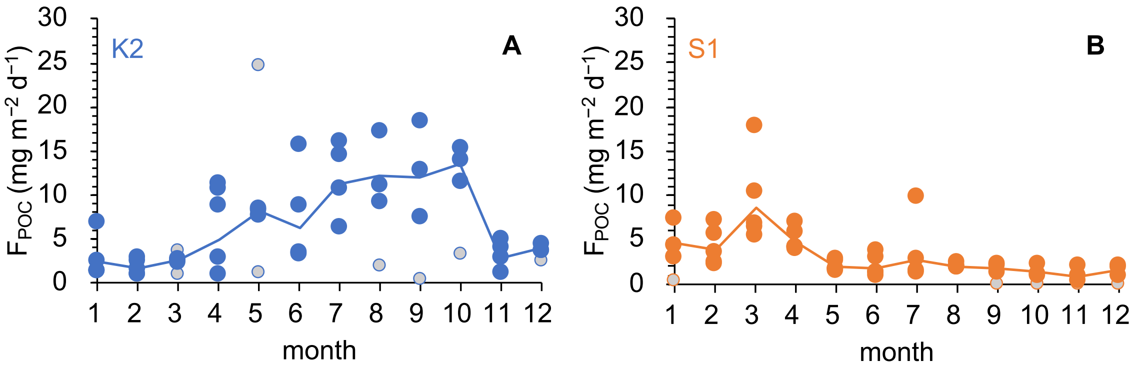


**Supplementary Figure S2.** Variations in particulate organic carbon flux ($F_{POC}$) at 500 m in K2 (A) and S1 (B), obtained from sediment trap deployments during 2010–2014. Solod lines connect monthly means for the entire deployments. Gray circles in both panels indicate monthly outliers defined using the 1.5-fold interquartile range rule for ln $F_{POC}$, the log-transformed of $F_{POC}$ (these outliers are not included in the calculation of monthly means).

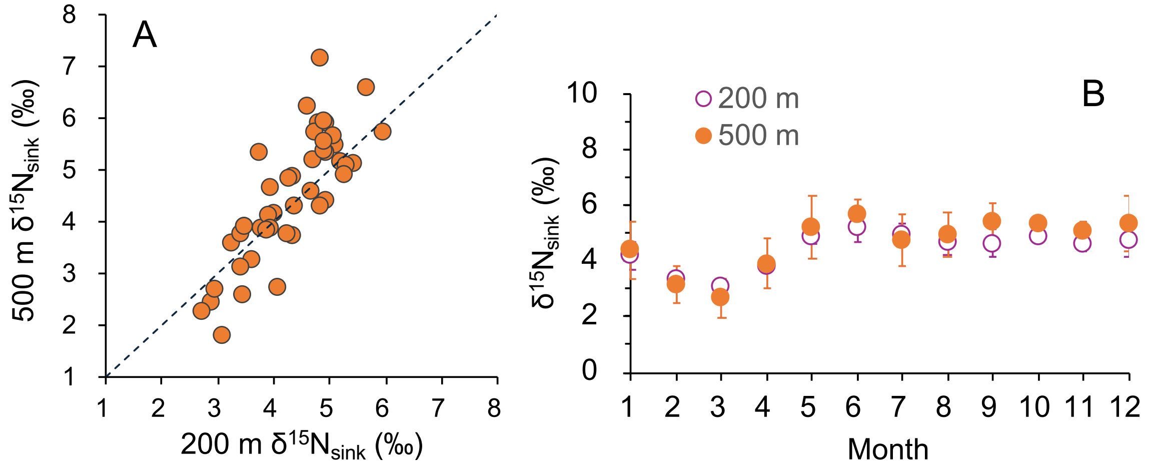


| $\delta^{15}N_{sink}$ | 200 m | 500 m |
|---|---|---|
| n | 53 | 45 |
| Min. ‰ | 2.7 | 1.8 |
| Max. ‰ | 5.9 | 7.1 |
| Ave. ‰ | 4.4 ± 0.8 | 4.5 ± 1.2 |

**Supplementary Figure S3.** (A) Relationship between nitrogen stable isotopic composition of trapped particles ($\delta^{15}N_{sink}$) trapped at depths of 200 m and 500 m in S1 during 2010–2014. Statistical measures for both datasets are listed below. (B) Variations in monthly mean $\delta^{15}N_{sink}$ for the entire deployments. Open circles indicate 200 m data; filled circles indicate 500 m data. Vertical bars indicate standard deviation. Note that the 200 m data used here have been published in Mino et al. (2020).

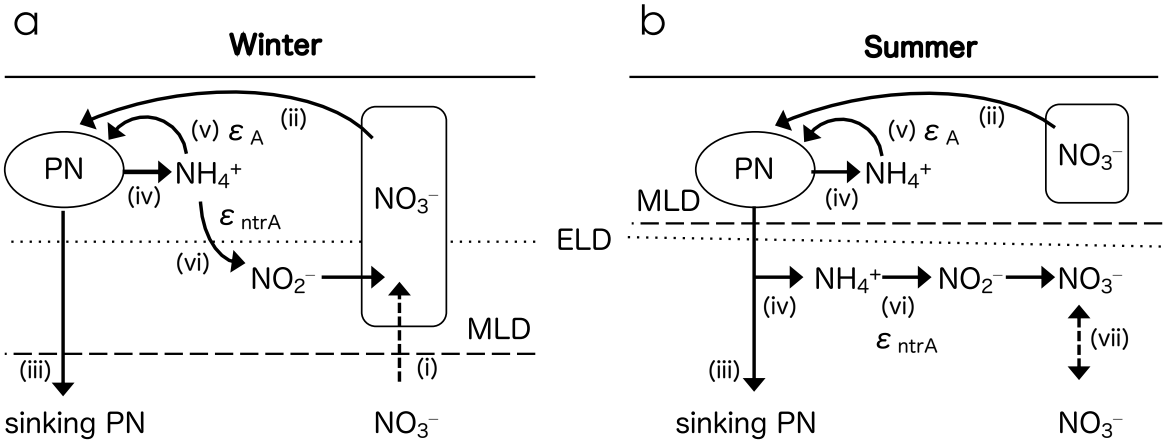


**Supplementary Figure S4.** Simplified nitrogen cycling in the upper water column during (a) winter and (b) summer at the station K2. Arrows indicate fluxes: (i) $NO_3^-$ transport due to winter convection, (ii) $NO_3^-$ uptake by phytoplankton, (iii) PN sinking, (iv) Regeneration of $NH_4^+$ (ammonification by heterotrophs), (v) $NH_4^+$ uptake by phytoplankton, (vi) $NH_4^+$ oxidation by nitrifiers, (vii) $NO_3^-$ diffusion. Dotted and dashed lines indicate depths of the euphotic layer (ELD) and the mixed layer (MLD), respectively. For fluxes (v) and (vi), the isotopic fractionations associated with them are described as $\varepsilon_A$ and $\varepsilon_{ntrA}$, respectively. All nitrogen within the mixed layer ultimately originates from deep water $NO_3^-$ supplied by wintertime vertical mixing (i). This preformed nitrate is consumed by phytoplankton (ii) to form particulate nitrogen. Part of PN is removed as sinking PN from the mixed layer (iii). Another part is re-mineralized to ammonium by microbial ammonification (iv). Ammonium can then be reassimilated into the PN pool by algal uptake in the euphotic layer (v) or oxidized by nitrifiers to nitrite (vi), and further oxidized to nitrate in the aphotic layer. Here we assume that $NH_4^+$ oxidation by nitrifiers in the euphotic layer is suppressed due to the light inhibition (Olson, 1981) and the competition with phytoplankton. The balance of these fluxes and the isotopic fractionations associated with them determine the $\delta^{15}N$ of each nitrogen pool. As for the ammonium pool in summer (panel b), it is regulated by both regeneration (iv) and reassimilation (v) of $NH_4^+$ in the shallow mixed layer. Below the euphotic layer, $NH_4^+$ oxidation (vi) occurs but does not affect the ammonium pool within the upper mixed layer (and the euphotic layer). On the other hand, in winter (panel a) nitrification does affect the ammonium pool since the MLD is deeper than the ELD. The isotopic fractionation during ammonium oxidation ($\varepsilon_{ntrA}$: –38‰, Casciotti et al. 2003) is larger in magnitude than the isotopic fractionation during assimilation ($\varepsilon_A$: –27‰, Waser et al. 1998). This can make the residual ammonium pool more enriched in $^{15}N$ in winter relative to that in summer. Reproduced from Mino et al. (2016).

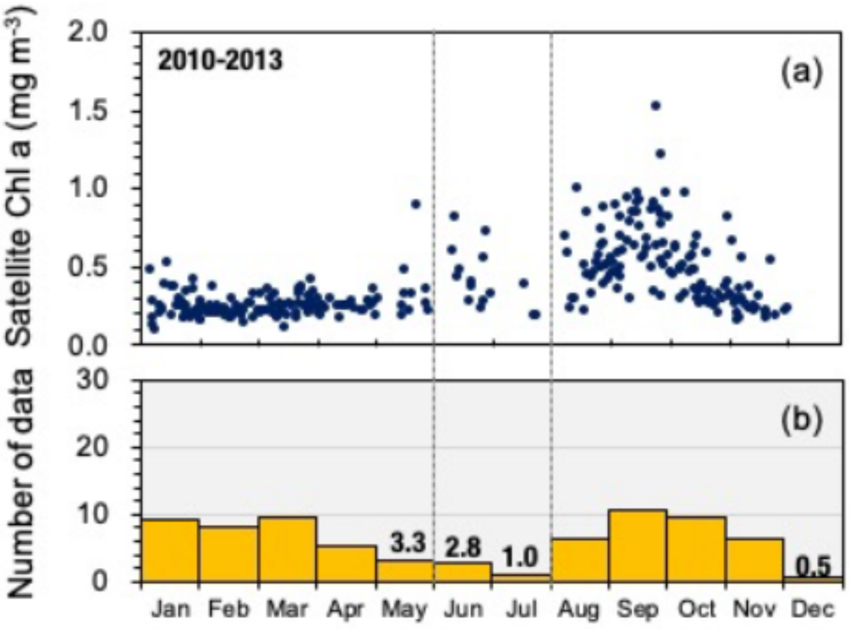


**Supplementary Figure S5.** (a) Time series of MODIS satellite surface chlorophyll a at K2 and (b) mean number of daily data obtained in each month during 2010–2013. The number less than 5 was denoted. Value was based on an areal average over an 11 x 11 pixel (approximately 100 x 100 km) square box. Data was obtained when more than one pixel was available. Due to frequent dense fog in the summer (Sasakawa et al. 2003), data acquisition is very limited in the Western Subarctic Gyre.

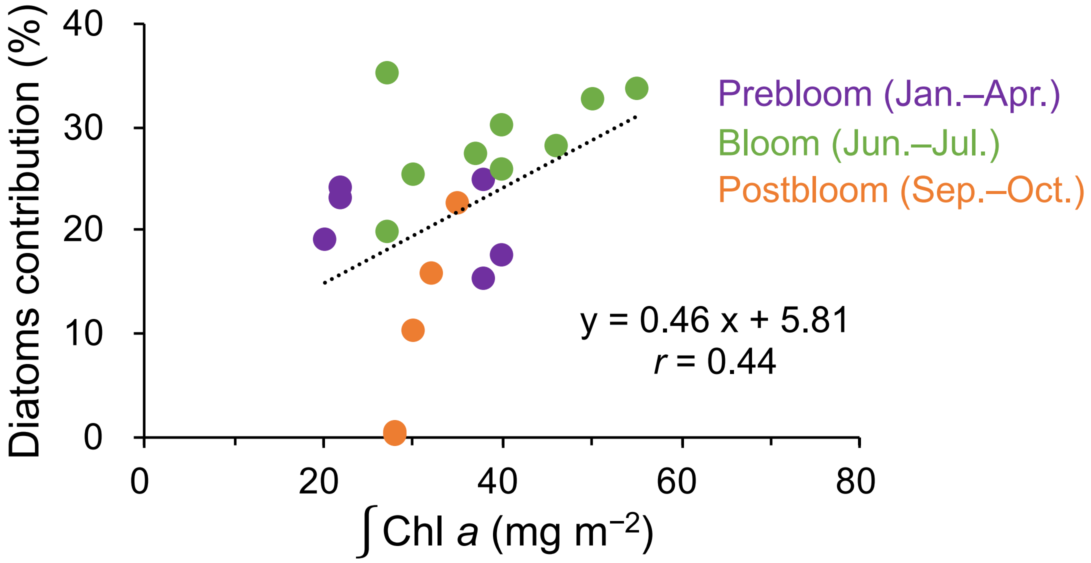


**Supplementary Figure S6.** Relationship between depth-integrated chlorophyll *a* (∫Chl *a*) and the diatom contribution at K2, based on cruise observations conducted from 2006 to 2012. The dotted line indicates the regression line for all data ($p = 0.05$). Diatom contributions were estimated from chemotaxonomic pigment concentrations using the CHEMTAX program. The ∫Chl a and diatom contribution data were taken from Fujiki et al. (2014); analytical details are provided therein.

**References:**


Fujiki, T., Sasaoka, K., Matsumoto, K., Wakita, M., & Mino, Y. Seasonal variability of phytoplankton community structure in the subtropical western North Pacific. *Journal of Oceanography* ***72***, 343–358 (2016).

Mino, Y. et al. Seasonal variations in the nitrogen isotopic composition of settling particles at station K2 in the western subarctic North Pacific. *Journal of Oceanography* ***72***, 819–836 (2016).

Mino, Y. et al. Seasonal and interannual variations in nitrogen availability and particle export in the northwestern North Pacific subtropical gyre. *Journal of Geophysical Research: Oceans* ***125***, e2019JC015600 (2020).

Olson, R. Differential photoinhibition of marine nitrifying bacteria: a possible mechanism for the formation of the primary nitrite maximum. *Journal of Marine Research* ***39***, 227–238 (1981).

Sasakawa, M., Ooki, A., & Uematsu, M. Aerosol size distribution during sea fog and its scavenge process of chemical substances over the northwestern North Pacific. *Journal of Geophysical Research: Atmospheres* ***108(D3)***, 1–9 (2003).

Waser, N. A. D., Harrison, P. J., Nielsen, B., & Calvert, H. E. Nitrogen iso- tope fractionation during the uptake and assimilation of nitrate, nitrite, ammonium and urea by a marine diatom. *Limnology and Oceanography* ***43***, 215–224 (1998).
